# Chemotherapy-mediated miR-29b expression inhibits the invasion and angiogenesis of cervical cancer

**DOI:** 10.18632/oncotarget.14738

**Published:** 2017-01-19

**Authors:** Yunyun Li, Zhongzu Zhang, Zhenghua Xiao, Ying Lin, Tangshu Luo, Qin Zhou, Xiaojing Zhang

**Affiliations:** ^1^ Department of Gynecology and Obstetrics, The Yongchuan Hospital of Chongqing Medical University, Chongqing 402160, PR China; ^2^ Department of Orthopedics, The Yongchuan Hospital of Chongqing Medical University, Chongqing 402160, PR China; ^3^ Department of Gynecology and Obstetrics, The First Affiliated Hospital of Chongqing Medical University, Chongqing 400016, PR China

**Keywords:** cervical cancer, miR-29b, STAT3, chemotherapy, EMT

## Abstract

Radiotherapy combined with platinum-based chemotherapy is the standard-of-care of locally advanced cervical cancer (CC) patients, while nearly 50% of patients do not respond to standard chemotherapy. Thus, identification of relative molecules participated in chemotherapy might provide new insights in the treatment of CC. In this study, we found a cohort of miRNAs were dysregulated upon treatment with cisplatin, among of which miR-29b was the most upregulated one. We further detected its expression in CC tissues, and found that miR-29b was significantly suppressed in CC and its precancerous lesions, HSIL tissues, and was negatively related with tumor invasion. However, upon treatment with cisplatin, the expression of miR-29b was significantly up-regulated. The biological function assays showed that overexpression of miR-29b suppressed the invasion, EMT procedure and angiogenesis of cervical cancer cells *in vitro* and inhibited tumor growth and neovascularization *in vivo* through targeting STAT3 signal pathway. While, inhibition of miR-29b could prevent the cisplatin-induced epithelial features, cell movement and angiogenesis of CC cells, which means miR-29b/STAT3 axis participates in the chemotherapy of cisplatin in CC. Collectively, our data suggest that chemotherapy-mediated miR-29b expression participates in the initiation and progression of cervical cancer through suppressing the proliferation, EMT procedure and angiogenesis of cervical cancer cells by targeting STAT3 signal pathway.

## INTRODUCTION

Cervical cancer (CC) is the second most common cancer in women worldwide. Despite the introduction of vaccines to high-risk human papilloma viral (hrHPV) strains, CC remains a major source of cancer related death, particularly in under-developed countries [1, 2]. The standard-of-care of locally advanced CC patients (stages IB2 to stages IVA according to FIGO) is surgery and/or radiotherapy (RT) in combination with chemotherapy. The addition of platinum-based chemotherapy to RT could significantly reduce the risk of death and disease progression for the early stage CC patients with risk factors for recurrence [3]. However, the intrinsic molecular mechanisms of chemotherapeutic drugs used for cervical cancer treatment are not well established.

MicroRNAs (miRNAs), a kind of small, non-coding RNAs, are considered to be important components of cancer signaling network and are emerging as novel biomarkers of diseases [4, 5]. Through partially complementing with the 3’-untranslated region of relative mRNAs, miRNAs modulate putative target gene expression by suppressing the translational efficiency or cleavage of target mRNAs [6]. The disruption of homeostasis in the miRNA/mRNA axis would lead to relevant pathologic events. Recently, the pivotal role of miRNAs in the chemotherapy of variety of cancers have been widely reported, such as miR-135a promotes the gastric cancer resistance to oxaliplatin through suppressing the E2F1/Sp1/DAPK2 pathway signaling [7]; overexpression of miR-126 could increase chemosensitivity in drug-resistant gastric cancer cells by targeting EZH2 [8]. For cervical cancer, through comparing the miRNAs profile between the non-response and complete-response patients upon chemotherapy with cisplatin, Abraham *et al*. identified a molecular signature consisting of seven validated miRNAs [9], while whether these miRNAs are all involved in the chemotherapy of cisplatin in cervical cancer, and the intrinsic mechanisms involved are less known.

In this study, we profiled the cervical cancer cells upon treatment with cisplatin, and found that miR-29b was significantly up-regulated upon treatment with cisplatin. Further biological experiments showed that overexpression of miR-29b not only suppressed the proliferation, metastasis and EMT procedure of cervical cancer cells, but also inhibited its angiogenesis, which suggests its suppressive effects in the chemotherapy of cisplatin in cervical cancer.

## RESULTS

### miR-29b expression is activated by cisplatin in cervical cancer cells

**Figure 1:**
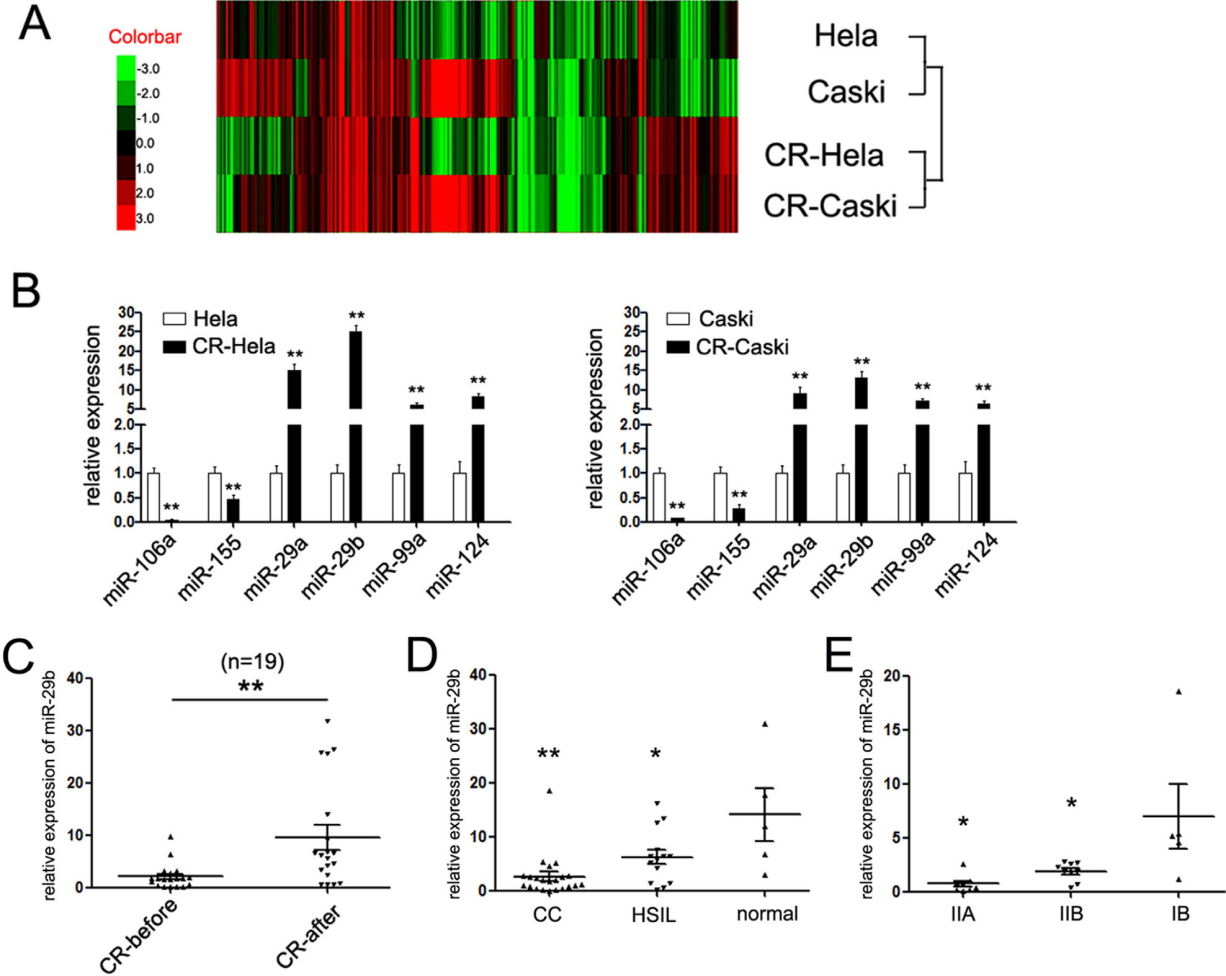
miR-29b was up-regulated upon treatment with cisplatin (**A**) Heat-map showing the expression profiles significantly differentially expressed in HeLa and Caski cells upon treatment with miR-29b. (**B**) The expression of six miRNAs in HeLa and Caski cells upon treatment with cisplatin was detected by quantitative RT-PCR. Data are shown as log10 of relative ratio change of the miRNAs relative to GAPDH; (**C**) Statistical analysis of relative mean level of miR-29b expression in 19 paired of cervical cancer tissues before and after chemotherapy with cisplatin; The expression of miR-29b was significantly increased upon treatment with cisplatin. (**D**) Statistical analysis of relative mean level of miR-29b expression in cervical cancer, HSIL tissues and normal tissues. Statistical analysis of relative mean level of miR-29b expression in a cohort of 22 cases of cervical cancers, 10 cases of high-grade squamous intraepithelial lesions (HSIL) and 5 cases of normal cervical tissues. The expression of miR-29b was suppressed in CC and HSIL tissues compared to normal tissues. (**E**) Statistical analysis of relative mean level of miR-29b expression in CC patients with different clinical stages. The expression of miR-29b was suppressed in later stages (stages IB compared with stages IIA and IIB). The data are representative of at least three independent experiments. Error bars represent s.e.m. **P < 0.01.

To identify putative miRNAs participating in the chemotherapy of cervical cancer, we analyzed the miRNAs expression profile in two cervical cancer cells (HeLa and Caski) upon treatment with cisplatin. Before the miRNA array profile, we treated HeLa and Caski cells with cisplatin (named CR-HeLa and CR-Caski cells, respectively), and estimated relative IC_50_ values of each cell lines (Supplementary Figure 1). The IC_50_ values were (14.42 ± 3.34) μM and (23.65 ± 1.83) μM in HeLa and Caski cells, respectively. Upon treatment, a total of 20 differentially expressed miRNAs were detected in cisplatin-treated cervical cancer cells, including 6 downregulated miRNAs and 14 upregulated miRNAs (Figure 1A, the detailed miRNAs were supplied in Supplementary Table 2). To further validate the miRNA array chip results, we selected 6 miRNAs (two downregulated miRNAs, miR-106a and miR-155, and 4 upregulated miRNAs, miR-29a/29b, miR-99a and miR-124) to determine their expression by qRT-PCR assays. The results were consistent with the miRNA array experiments (Figure 1B). Among of which, miR-29b was the most differently expressed miRNA detected, with more than twenty-five-fold and fifteen-fold augmented level upon treatment with cisplatin in HeLa and Caski cells, respectively. Since miR-29 family was reported to be associated with the chemotherapy in many cancers, we hypothesized that miR-29b might perform a prominent role in the chemotherapy of cervical cancer.

Thus, we further detected the expression of miR-29b in a cohort of 19 paired of cervical cancer tissues before and after chemotherapy of cisplatin. As expected, the expression level of miR-29b in CC patients after chemotherapy of cisplatin was much higher than which in the untreated group (Figure 1C). These results proposed that miR-29b-mediated program was involved in the chemotherapy with cisplatin, which might hold clinical promise for CC patients who are resistant for cisplatin.

### miR-29b was suppressed in cervical cancer tissues and negatively related to tumor invasion

We next examined the correlation between miR-29b expression and cervical cancer development. qRT-PCR assays were performed to assess miR-29b level in a cohort of 22 cases of cervical cancers, 10 cases of high-grade squamous intraepithelial lesions (HSIL) and 5 cases of normal cervical tissues. As shown in Figure 1D, the expression of miR-29b was significantly suppressed in cancer and HSIL tissues compared with the normal tissues, while no statistically difference were found in the expression of miR-29b between the cancer and HSIL tissues.

Moreover, the pathological and clinical characteristics of the 22 cases of cancer patients, including patients’ age at diagnosis, tumor size, whether infected with HPV-16 type and clinical stages were collected and supplemented in Table 1. The expression of miR-29b showed significant association with clinical stages, as tumors with latter stages (stage IIA and IIB) expressed lower level of miR-29b (Figure 1E). However, no statistical correlations were observed between the miR-29b expression with age, tumor size and HPVs infected subtype (Table 1). These results demonstrated that miR-29b might be involved in the cervical cancer invasion.

**Table 1:** Relationship between miR-29b expression and their clinicopathological parameters in cervical cancer patients

Clinicolpathological parameters	Number of cases	Expression of miR-29b	*P*-value
Age (years):			
< 40	15	2.678 ± 1.185	0.918
≥ 40	7	2.486 ± 0.768	
Histology:			
Squamous-cell carcinoma	18	2.638 ± 1.003	0.958
Adenocarcinoma	4	2.520 ± 1.009	
Tumor size before chemotherapy (cm):			
< 4	16	3.095 ± 1.114	0.360
≥ 4	6	1.341 ± 0.528	
Nodal status:			
Positive	20	2.268 ± 0.388	0.898
Negative	2	2.651 ± 0.917	
Clinical stages:			
Stage IB	5	6.984 ± 3.003	0.002^*^
Stage IIA and IIB	17	1.332 ± 0.243	
HPV types:			
HPV 16	19	2.594 ± 0.949	0.947
Other types	3	2.760 ± 1.386	

Abbreviations: *P*-value represents the probability from a Student's *t*-test for miR-29b expression between variable subgroups. *P < 0.05 and **P < 0.01 were considered to have a significant difference.

### Ectopic expression of miR-29b inhibits proliferation and invasion of cervical cancer cells *in vitro*

**Figure 2:**
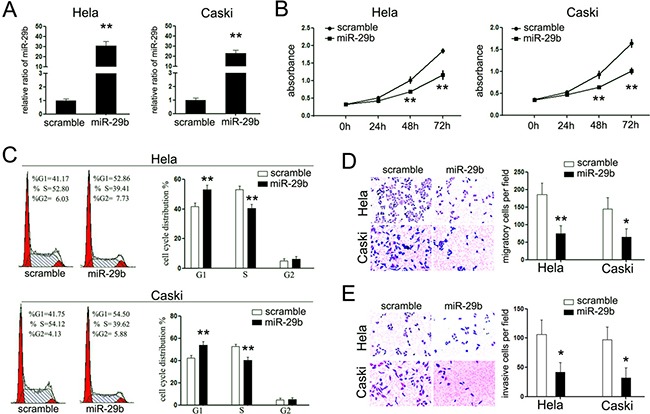
miR-29b functions as a tumor suppressor in cervical cancer (**A**) RT-PCR was performed to detect the expression of miR-29b in cervical cancer cells upon transfection with miR-29b mimic; (**B**) CCK-8 assays were performed to analyze the effect of miR-29b on cell proliferation of both cell lines. Overexpression of miR-29b suppressed the cell proliferation of both cell lines; (**C**) Cell cycle analysis, by fluorescence-activated cell sorting (FACS) at 48h after transfection, were performed to analyze the effect of miR-29b on cell cycle progression of both cell lines; (**D**, **E**) The effects of miR-29b on cell migration and invasion were detected using trans-well chamber assays. Panel D showed the results on migration (×400); Panel E showed the results on invasion (×400). The data are representative of three independent experiments. Error bars represent s.e.m. *P < 0.05; **P < 0.01.

From these analyses, we postulated that miR-29b might perform as a tumor suppressor in cervical cancer. To test this hypothesis, miR-29b overexpression models were established in cervical cancer cell lines, HeLa and Caski cells, by transiently transfection with miR-29b mimic (Figure 2A). Then, we investigated the effects of miR-29b overexpression on the proliferation rate of both cell lines. As shown in Figure 2B, overexpression of miR-29b suppressed the cell proliferation rates of both cells, and resulted in a significant increased number of cells in the G1-phase of cells cycle as well as a reduced amount of S-phase cells (Figure 2C). The suppression of miR-29b in the latter clinical stages of CC patients promoted us to examine its effects on the invasive capacity of HeLa and Caski cells. Transient miR-29b overexpression significantly inhibited migration and invasion of both HeLa and Caski cells as assessed by trans-well matrigel migration and invasion assays, respectively (Figure 2D and 2E). Overexpression of miR-29b significantly inhibited cells passing through the trans-well chambers in both experiments. These results further supported our proposition that miR-29b performs as a tumor suppressor in cervical cancer.

### Inducible miR-29b expression inhibits the EMT procedure and angiogenesis of cervical cancer

**Figure 3:**
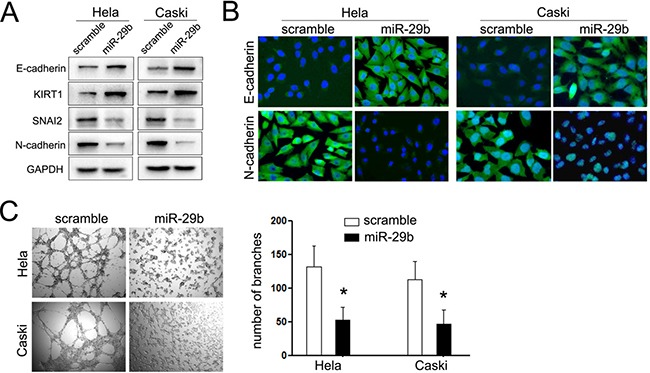
miR-29b suppressed the EMT procedure and angiogenesis of CC (**A**) Western blot analysis of the expression of epithelial and mesechymal marker proteins in cervical cancer cells transfected with miR-29b mimic was performed. (**B**) Immunofluorescence staining assays were further used to detect the expression of E-cadherin and N-cadherin in cervical cancer cells. The expression of N-cadherin was suppressed in cells transfected with miR-29b, while the expression of N-cadherin was increased in miR-29b group. (**C**) Vasculogenic mimicry (VM) assays were performed to explored the effects of miR-29b on the angiogenesis of CC cells. Left panel showed the represent images of vasculogenic mimicry; right panel showed the statistical significance of experiments. The data are representative of three independent experiments. Error bars represent s.e.m. *P < 0.05; **P < 0.01.

The suppressive effects of miR-29b on the invasion of cervical cancer made us speculate that whether it performs impacts on the EMT procedure and angiogenesis of cervical cancer, since both of which perform pivotal role in tumor invasion and metastasis. As expected, cells overexpressing miR-29b increased the expression of epithelial markers (E-cadherin and KIRT1), and decreased the expression of mesenchymal markers (N-cadherin and SNAI2) (Figure 3A). The same results were further identified by immunofluorescence staining assays. The expression of epithelial marker, E-cadherin was induced and localized to the plasma membrane in miR-124 treated cells. On the opposite, the mesenchymal marker N-cadherin expression was notably decreased (Figure 3B).

The next important steps for tumor invasion are the degradation of basal membranes and the extracellular matrix (ECM), and formation of vasculogenic mimicry (VM), which would provide the nutrition for the tumors cells in the central. The former has been identified using the Matrigel chamber Transwell assays, which showed overexpression of miR-124 inhibited cells passing through the chambers coated with Matrigel. Thus, we further explored the effects of miR-29b on formation of VM using tube-like channel formation assays. As expected, overexpression of miR-29b decreased approximately 50% and 48% of the tube-like channels in HeLa and Caski cells, respectively (Figure 3C). These results demonstrated that miR-29b not only performs suppressive effects on the migratory and invasive capacity of cervical cancer cells, but also inhibits its EMT and angiogenesis procedure.

### miR-29b inhibits STAT3 expression through binding to its 3′UTR

**Figure 4:**
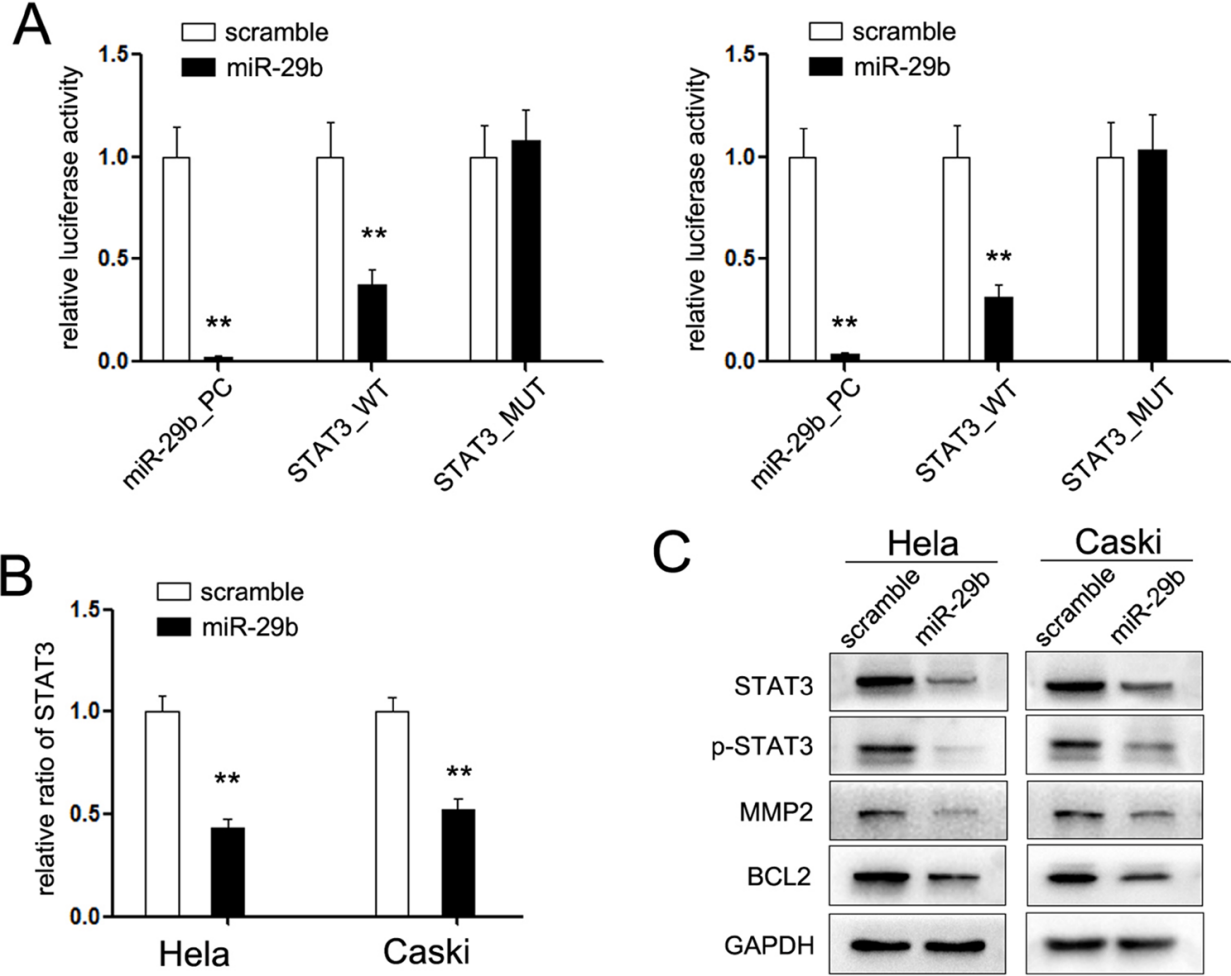
STAT3 is the target gene of miR-29b (**A**) Relative luciferase activity of the indicated STAT3 reporter construct in cervical cancer cells, co-transfected with miR-29b mimic or scramble mimic, is shown; (**B**) Quantitation RT-PCR of the expression level of STAT3 mRNA in cervical cancer cells treated with miR-29b mimic; (**C**) Western blot analysis showed the expression level of STAT3 and its downstream protein in CC cells treated with miR-29b mimic.

Although miR-29b tunes the expression of variety genes, its mechanisms mediated in the regulation of cervical cancer cells remain less known. Previous studies have indicated that miR-29b directly targets signal transducer and activator of transcription 3 (STAT3), which perform a pivotal role in the cellular signal pathway. To comfier whether the miR-29b/STAT3 axis performs function in cervical cancer, we analyzed the binding ability of miR-29b to wild or mutant STAT3 3′UTR using the dual-luciferase reporter assays. Our results indicated that miR-29b overexpression partly inhibited the transcriptional activity of luciferase reporter containing mutant STAT3 3′UTR construct, but did not alter the luciferase activity of the mutated reporter construct lacking the miR-29b binding site (Figure 4A). Restored the expression of miR-29b reduced the mRNA and protein level of STAT3 in HeLa and Caski cervical cancer cells (Figure 4B and 4C). Moreover, the active form of STAT3, phospho-STAT3 (p-STAT3), and two identified downstream genes of STAT3, BCL-2 and MMP-2, were consistently suppressed (Figure 4C), which demonstrated that STAT3 is a target gene of miR-29b in cervical cancer cells.

### Drug treatment of cisplatin suppresses cervical cancer cell movement and angiogenesis via regulating miR-29b/STAT3 axis

**Figure 5:**
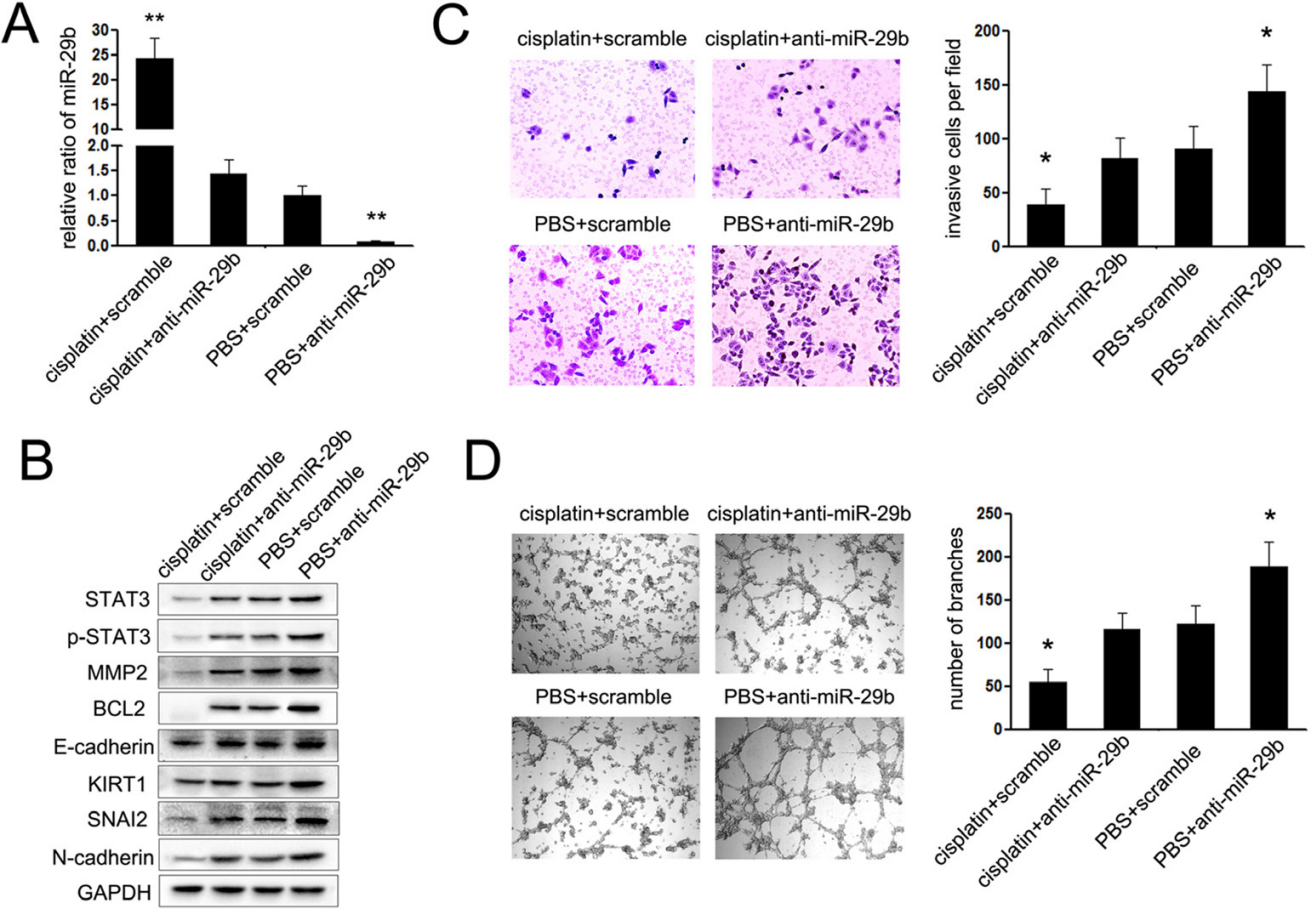
miR-29b/STAT3 axis was involved in the chemotherapy of cisplatin (**A**) The expression of miR-29b in cervical cancer cells transfected with anti-miR-29b or scramble mimic after treated with cisplatin or PBS; (**B**) Western blot assays were performed to explored the expression of STAT3, p-STAT3, MMP2, BCL2, E-cadherin, KIRT1, SNAI2 and N-cadherin in cervical cells showed in A; (**C**) Matrigel invasion assays were performed to explore the effects of miR-29b on the invasion of CC cells showed in A; (**D**) vasculogenic mimicry (VM) assays were performed to explored the effects of miR-29b on the angiogenesis of CC cells showed in A. The data are representative of three independent experiments. Error bars represent s.e.m. *P < 0.05; **P < 0.01.

To investigate whether cisplatin represented its suppressive effects on cervical cancer *via* regulating miR-29b/STAT3 axis, rescue assays were performed. In doing so, inhibitor for miR-29b (anti-miR-29b) was transfected into HeLa and Caski cells upon treatment with cisplatin to re-modify the miR-29b-mediated STAT3 axis. As shown in Figure 5A, cisplatin treatment led to an increase of miR-29b expression which was further dampened by anti-miR-29b transfection. We also observed that STAT3 protein as well its downstream genes, BCL2 and MMP2, were all decreased upon drug treatment and increased with anti-miR-29b transfection (Figure 5B). The following immunoblot and invasion assays indicated that miR-29b inhibition to prevent its induction by cisplatin treatment led to increased epithelial phenotype and cell movement (Figure 5B and 5C). Lastly, the similarity was also found in vasculogenic mimicry assays, as anti-miR-29b significantly restored the suppressive effects of cisplatin on vasculogenic mimicry of HeLa and Caski cells (Figure 5D). These findings suggest that the miR-29b-mediated STAT3 axis is a target of cisplatin treatment in cervical cancer cells.

### Ectopic expression of miR-29b impairs the xenografts formation of HeLa cells *in vivo*

**Figure 6:**
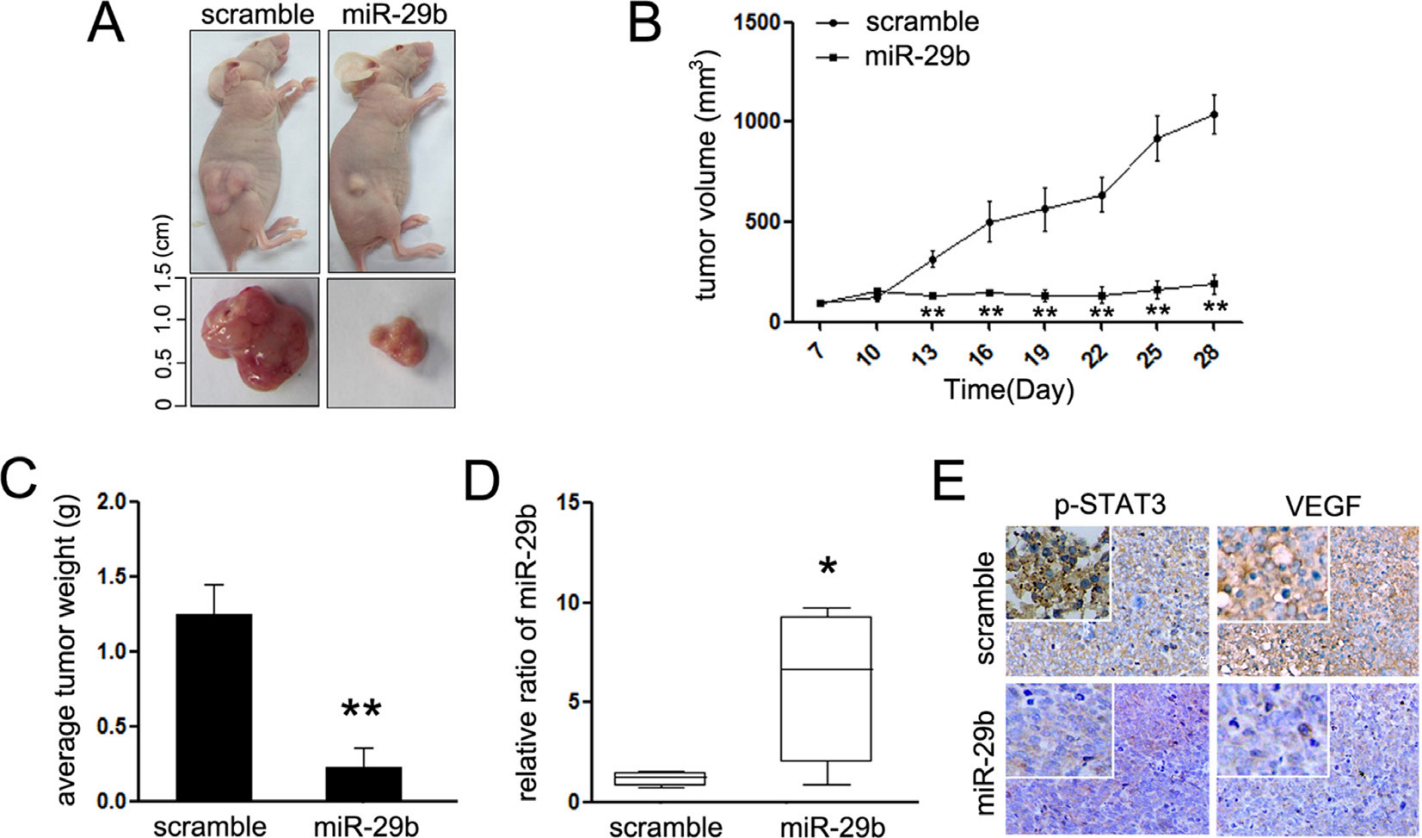
MiR-29b inhibits cervical cancer growth *in vivo* (**A**) Graphic representing tumor volumes at the end of the experiment for mice treated with miR-29b mimic or scramble mimic. Five mice per group; (**B**) Tumor volume averages between scramble and miR-29b mimic treated mice groups at the indicated days during the experiment; (**C**) Tumor weight averages between scramble and miR-29b mimic treated mice groups at the end of the experiment (28 days); (**D**) Quantitative RT-PCR analysis showed the relative expression of miR-29b in injected tumor tissues (normalized to U6); (**E**) Representative images of the immunohistochemistry analysis of p-STAT3 and VEGF in tumors from xenograft mice. Data are presented as means ± SD. **P < 0.01.

To validate the function of miR-29b *in vivo*, miR-29b and relative scramble mimic were used to treat the mice xenograft models where approximately HeLa cells were subcutaneously injected into the immunocompromised mice (Figure 6A). MiR-29b induced a slight inhibition of tumor growth (Figure 6B and 6C), resulting in a significantly reduction in the average tumor volume and weight (Figure 6D). RT-PCR was adopted to confirm the expression of miR-29b in HeLa cells from the tumor masses. As expected, miR-29b was upregulated in the tumor masses (Figure 6D), which demonstrated that the treatment is effective. Moreover, the expression of p-STAT3 and VEGF, which present the formation of vessel in tumor mass, was significantly suppressed in miR-29b-injected tumors (Figure 6E). These results indicate that miR-29b reduces mouse tumor growth and vasculogenic mimicry of cervical cancer *in vivo*, and the STAT3 mediated signaling pathway was involved in its suppressive effects.

## DISCUSSION

Cisplatin is a front-line chemotherapeutic agent for treating cervical cancer. Unfortunately, nearly 50% of CC patients do not respond to the standard chemotherapy, and these patients have a high recurrence rat and worse survival in the first five years [10]. Thus, understanding the putative mechanisms involved in chemotherapy of cisplatin might provide new insights in the treatment of cervical cancer, especially the one with chemoresistance or non-response. In this study, we reported a cohort of miRNAs dysregulated upon treatment with cisplatin, from which miR-29b was validated to be correlated with cisplatin chemotherapy, and performed as a tumor suppressor through suppressing the invasion and angiogenesis of cervical cancer cells.

MiR-29b belongs to the miR-29s family, which was a subclass of “epi-miRNAs”, as they target a subset of epigenetic regulators such as DNA methyltransferases (DNMTs), histone deacetylases (HDACs), and revert aberrant epigenetic alterations in cancers [11]; For example miR-29b could turn off the synthesis of key OBL-inhibitor proteins, TGFβ3 and HDAC4, allowing the expression of Runx2 and suppression of the Wnt pathway inhibitor catenin beta interacting protein 1 (CTNNBIP1), thus contributes to the osteoblast differentiation [12]. However, the putative effects of miR-29b in cervical cancer is still less known. Herein, we found that miR-29b was suppressed in cervical cancer tissues and its precancerous lesions, HSIL tissues. These results are partially consistent with Li *et al*., who reported that miR-29a was involved in HPV infection, and promoted malignant transformation of CC cells through targeting YY1 and CDK6 [13]. They also found that miR-29a was significantly suppressed in cervical cancer tissues, while they did not found any differences between the HSIL and normal tissues. Thus, they proposed that aberrant expression of miR-29a did not occur at the initial stage of HPV infection prior to the morphology change. The difference might due to the different HPV infection status between two sample groups. Although we did not identify the putative relation between miR-29b expression and HPV infection, we found that miR-29b was involved in chemotherapy of cisplatin, as treatment with cisplatin could up-regulate the expression of miR-29b in cervical cancer cells and tissues.

To further identify the mechanisms of miR-29b involved in chemotherapy, we detected the effects of miR-29b on the biological function of cervical cancer cells. As expected, overexpression of miR-29b significantly suppressed the cell proliferation and cell cycle progression of both cervical cancer cells. On the other hand, since the expression of miR-29b was related to the clinical stages of cervical cancer, we proposed that miR-29b might participate in the regulation of tumor invasion. Not only the suppression on the migratory and invasive capacity, but also rearrangements from a mesenchymal to an epithelial-like state were found in cervical cancer cells transfected with miR-29b. Tumor cells that undergo the EMT procedure which makes cells gain a fibroblast-like elongated morphology, and lose their cell-cell contacts, were more resistant to chemotherapy, and these cells have undergone mesenchymal-to-epithelial transition (MET) in the metastatic organs [14]. For cervical cancer, EMT has been reported to be associated with greater tumor invasion and progression, and be associated with the expression of EGFR, which plays a vital role in chemo and radiotherapy responses [14]; Herein, we also found that miR-29b could reverse the EMT procedure and inhibits the angiogenesis of cervical cancer cells *in vitro* and *in vivo*, and miR-29b inhibition could prevent the induction of cisplatin treatment to epithelial phenotype and cell movement, as well as vasculogenic mimicry formation. These data suggested that miR-29b participated in the chemotherapy of cisplatin through regulating the EMT procedure and angiogenesis of cervical cancer cells. Thus, administration of miR-29b mimic to cervical cancer patients may provide a novel therapy to block cervical carcinogenesis and progression.

Lastly, to further figure out the putative mechanisms involved in miR-29b-mediated suppressive effects, the target genes of miR-29b were searched. Variety of oncogenes have been reported to be target genes of miR-29b, among of which, STAT3 attracted our attention most. STAT3 is a critical member of signal transducer and activator of transcription (STAT) protein family. By modulating the transcription of many genes that regulate a wide variety of biological processes, including cell growth, apoptosis, immune response and angiogenesis, the STAT3 oncoprotein drives tumorigenesis in many tissue types, especially in the gynecology tumors as we previously mentioned [5, 15]. We identified that STAT3 was a functional target gene of miR-29b in cervical cancer cells, and might be involved in the miR-29b axis on cervical cancer chemotherapy of cisplatin. Except for STAT3, VEGF, which plays a pivotal role in promoting neovascularization, was reported to be another target gene of miR-29b in endothelial cells [16]. This might also explain the putative mechanisms involved in miR-29b-mediated suppression on neovascularization of cervical cancer. Moreover, cyclin-dependent kinase-6 (CDK6), an important cell cycle-related protein that regulates the phosphorylateion level of the downstream gene of p53, Rb [17], was also reported to be the target gene of miR-29b. Li Yang *et al*. proposed that miR-29b might participate in the cervical carcinogenesis through targeting YY1 and CDK6, since E6 and E7 oncoproteins encoded by HPV genome could deregulate the cellular proliferation and apoptosis through targeting p53 and pRb, respectively. This might also be another important mechanism involved in miR-29b-mediated cervical tumorigenesis, however, the exact mechanisms involved need more experiments to identify.

Taken together, our work demonstrated that chemotherapy with cisplatin-mediated expression of miR-29b functions as a tumor suppressor through targeting STAT3 signal pathway mediated suppression on proliferation, invasion, EMT procedure and angiogenesis of cervical cancer.

## MATERIALS AND METHODS

### Patients and tissue specimens

A total of 22 cases of cervical cancer, 10 cases of high-grade squamous intraepithelial lesions (HSIL) and 5 cases of normal cervical tissues were collected between 2014 and 2016 at Yongchuan Hospital of Chongqing Medical University. The tissues of cervical cancer patients were collected at the time of surgery and were confirmed by pathologists. Among the cancer tissues, only 19 paired of samples were collected when they were validated and before chemotherapy. The detailed pathological and clinical characteristics were supplied in Table 1. Upon resection, human surgical specimens were immediately frozen in liquid nitrogen and stored at –80°C in the refrigerator. The RNA of tissues was isolated from the paraffin tissues of biopsy. All patients did not undergo any therapy before recruitment to this research. Use of the tissue samples for all experiments was approved by Ethics Committee of the instruction.

### Cell culture and transfection

Human cervical caner cell lines (HeLa and Caski cells) were obtained from the American Type Culture Collection. These cell lines have been authenticated (Hybribio Bioscience & Technology Inc., Guangzhou, China). All cells were immediately expanded and frozen such that they could be restarted every 3 to 4 months from a frozen vial of the same batch of cells. HeLa and Caski cells were maintained in DMEM medium (PAA, Austria) supplemented with 10% fetal bovine serum (FBS; PAA, Austria), streptomycin (100 μg/ml), and penicillin (100 U/ml). All cells were incubated in a humidified atmosphere of 5% CO_2_ at 37°C as previously reported. MiR-29b/scramble mimic constructs were purchased from Dharmacon (Austin, TX, USA). According to manufacturer's instructions, all oligonucleotides were transfected into HeLa and Caski cells to a final concentration of 50 nM by Dhamafect 1 (Dharmacon, Lafayette, CO, USA). Cells were collected for further experiments 48 h post-transfection.

### Cell proliferation and FACS analyses

For cell proliferation analysis, HeLa and Caski cells were seeded into 24-well plates at 8 × 10^3^ cells per well, and then incubated in 10% Cell Counting Kit-8 buffer (CCK-8, Dojindo, Japan) diluted in normal culture medium at 37°C until visible color conversion occurred. The proliferation rate was determined 0, 24, 48, and 72 h after transfection. The absorbance in each well was measured at 450 nM and 630 nM using a microplate reader. To analyze the effects of cisplatin, cells were treated at concentrations of 0, 8, 16, 32, 64, 128 μM with cisplatin for 24 h. The IC_50_ value was calculated as the concentration of paclitaxel that reduced cell viability by 50%. For cell cycle analysis, cells were harvested 48 h after transfection, washed twice with cold PBS, fixed in ice-cold 70% ethanol, incubated with propidium iodide and RNase A, and then analyzed by fluorescence-activated cell sorting (FACS). All experiments were performed four times and the average percentages of cells are shown.

### Cell migration and invasion assays

Migration assays were carried out in modified Boyden chambers (BD Biosciences, San Jose, CA, USA) with 8 μm pore filter inserts in 24-well plates. 24 h after transfection, 2 × 10^5^ cells suspended in serum-free medium were added to the upper chamber. Medium containing 20% FBS were added to the lower chambers as a chemoattractant. After 24 h transfection, the non-filtered cells were gently removed with a cotton swab. Filtered cells located on the lower side of the chamber were stained with crystal violet, air-dried and photographed. For analysis of invasive capacity, the transwell migration chambers were coated with Matrigel (BD Biosciences, San Jose, CA, USA) and incubated at 37°C for 3 h, allowing it to solidify. After 24 h of transfection, 4 × 10^5^ cells suspended in serum-free medium were added to the upper chamber. The remaining steps were the same as migration assays. Three independent experiments were performed.

### RNA extraction and quantitative real-time PCR

For analysis the expression of relative miRNAs in cervical cancer, total RNA was isolated from human surgical specimens and cells according to the protocol of Recover All Total Nucleic Acid Isolation Kit (Ambion, Austin, TX, USA). Following gel electrophoresis verification of RNA integrity, total RNA was reverse transcribed using a First-Strand cDNA Synthesis kit (Invitrogen, Carlsbad, CA, USA) with specific primers. The expression of small nuclear U6 was used as internal control. Then, quantitative real-time PCR was performed to quantify relative expression of miRNAs using the Quanti-Tect SYBR Green PCR mixture on an ABI PRISM 7900 Sequence Detection System (Applied Biosystems, Carlsbad, CA, USA). For analysis of mRNA expression, the expression of GAPDH was used as internal control and Oligo (dT) was used as the primer for reverse transcription. PCR efficiencies were calculated wi#f6th a relative standard curve derived from a complementary DNA mixture and gave regression coefficients > 0.95. The relative expression levels were evaluated using the 2^−ΔΔ^C^t^ method. All experiments repeated four times. The primers mentioned above were summarized in Supplementary Table 1.

### Luciferase reporter assays

To further identify the target role of STAT3, the complete STAT3 3′UTRs were amplified from genomic DNA and cloned into the pGL-3-vector (Promega, San Luis, CA, USA), respectively. The QuickChange Site-Directed Mutagenesis kit (Stratagene, Santa Clara, CA, USA) was used to mutate the 3′UTRs of both target genes. The luciferase reporter construct containing the miR-29b consensus target sequence served as a positive control (PC). About 1 × 10^5^ cells per well were seeded into 24-well plates and grown for 24 h prior to transfection. Cells were transfected with the pGL-3 firefly luciferase reporter (50 ng per well), pRL-TK Renilla luciferase reporter (10 ng per well), and the miR-29b mimics (50 nM). The pRL-TK vector served as an internal control. All transfections were carried out in triplicate using Lipofectamine 2000 (Invitrogen, USA). Cell lysates were prepared using Passive Lysis Buffer (Promega, San Luis, CA, USA) 48 h after transfection, and luciferase activity was measured using the Dual-Luciferase Reporter Assay (Promega, San Luis, CA, USA) and normalized to Renilla luciferase activity.

### Westernblot analysis

For the westernblot assay, cells were harvested in ice-cold PBS 48 h after transfection and lysed on ice in cold-modified radioimmunoprecipitation buffer supplemented with protease inhibitors. Protein concentration was determined using the BCA Protein Assay Kit (Bio-Rad, CA, USA) and equal amounts of protein were analyzed by SDS-PAGE. Gels were electroblotted onto nitrocellulose membranes (Millipore, WI, USA). After blocked with 5% non-fat dry milk in Tris-buffered saline containing 0.1% Tween-20 2 h, membranes were incubated at 4°C over night with primary antibody. Primary antibodies used were anti-STAT3, anti-p-STAT3, anti-MMP2, anti-BCL2, anti-E-cadherin, anti-KIRT1, anti-SNAI2, anti-N-cadherin (Cell Signaling, USA) and GAPDH (Zhong-Shan JinQiao, China). Then, membranes were incubated with respective second antibodies and detected by peroxidase-conjugated secondary antibodies using the enhanced chemiluminescence system (ECL) (Millipore, WI, USA). The experiment was repeated three times.

### Vasculogenic mimicry formation assays

To test the effects of miR-29b on the formation of vasculogenic mimicry of cervical cancer cells, Matrigel was plated on the 24-well plate, and 5 × 10^5^ HeLa and Caski cells were seeded on it. Twelve hours after seeding, the tube-like structures were examined by light microscopy, photographed and counted.

### Immunofluorescent staining

Cervical cancer cells were seeded onto coverslips in six-well culture plates and transiently transfected with miR-29b or scramble mimic. Cells were fixed with 4% paraformaldehyde at room temperature for 20 min, washed in PBS, permeabilized with PBS containing 0.1% Triton X-100 (PBS-T) at room temperature for 90 min, and blocked with 1% BSA in PBS-T. Cells were then incubated with human anti-E-cadherin and anti-N-cadherin antibodies (1:200; Cell Signaling Technology Company) overnight at 4°C. The primary antibody was detected using anti-rabbit-Alexa 594-conjugated antibodies (1:200; Invitrogen). To detect nuclei, cells were co-stained with 4’, 6-diamidino-2-phenylindole. Fluorescence was observed and imaged using a Nikon Eclipse TE300 confocal laser microscope.

### *In vivo* assays

Five-week-old BALB/C-nu/nu nude male mice were used for animal studies, and all animals were maintained in the pathogen-free conditions at our institution. For animal xengraft model assays, ten mice were divided into two groups randomly. 2 × 10^6^ HeLa cells were subcutaneously injected into the posterior flanks of each mice. When tumors reached 50 mm^3^, miR-29b or scramble mimic (100 nmol) was suspended in Lipofectamine 2000 (100 μl) and was injected into the tumors directly every 3 days for a total of 7 times. Tumor diameters were measured after 7 days from injection and then every 3 days. At 28 days after injection, mice were killed and tumors were weighted after necropsy. Tumor volume was calculated as follows: length × width^2^ × 1/2. The mice xenograft model assays were performed according to institutional guidelines.

### Statistical analysis

Data were expressed as the mean ± standard deviation of at least three independent experiments. Statistical analysis was carried out using SPSS 15.0 software. The Student's t-test was used for comparisons between two groups, and analysis of variance was used for comparisons among three groups. The chi-squared test was used for occurrence analysis. *P* values of less than 0.05 were considered to be significant.

## SUPPLEMENTARY MATERIALS FIGURES AND TABLES



## Abbreviations

CC: cervical cancer
EMT: epithelial to mesenchymal transition
miRNAs: microRNAs
3’UTR: 3’-untranslated region
hrHPV: high-risk human papilloma viral
RT: radiotherapy
HSIL: high-grade squamous intraepithelial lesions
ECM: extracellular matrix
VM: vasculogenic mimicry
DNMTs: DNA methyltransferases
HDACs: histone deacetylases
CTNNBIP1: catenin beta interacting protein 1
MET: mesenchymal-to-epithelial transition
STAT: signal transducer and activator of transcription
CDK6: cyclin-dependent kinase-6
CCK-8: Cell Counting Kit-8
FACS: fluorescence-activated cell sorting
ECL: enhanced chemiluminescence system
