# Supplementary material for: Chemotherapy-mediated miR-29b expression inhibits the invasion and angiogenesis of cervical cancer
Source: Oncotarget. 2017 Jan 19;8(9):14655–65. doi: 10.18632/oncotarget.14738 (PMC5362433; doi:10.18632/oncotarget.14738)
Supplement: Supplementary file 1 [file oncotarget-08-14655-s001.pdf]

Chemotherapy-mediated miR-29b expression inhibits the invasion and angiogenesis of cervical cancer

Supplementary Materials

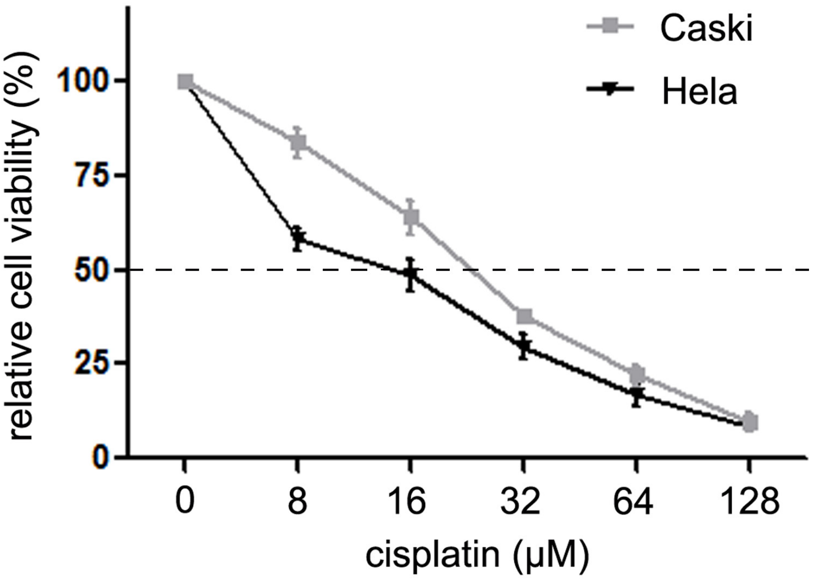

Supplementary Figure 1: The relative viability rate of cervical cancer cells upon treatment with different density of cisplatin. The IC50 values were (14.42 ± 3.34) µM and (23.65 ± 1.83) µM in HeLa and Caski cells, respectively.

Supplementary Table 1: Primer sequences for PCR or reverse transcription

Gene	Primer Sequence
Primers for Reverse transcription	
miR-29a	5'- GTCGTATCCAGTGCAGGGTCCGAGGTATTCGCACTGGTAACCG-3'
miR-29b	5'-GTCGTATCCAGTGCAGGGTCCGAGGTATTCGCACTGGAACACTG-3'
miR-124	5'-GTCGTATCCAGTGCAGGGTCCGAGGTATTCGCACTGGGGCATTC-3'
miR-155	5'-GTCGTATCCAGTGCAGGGTCCGAGGTATTCGCACTGGACCCCT-3'
miR-106a	5'- GTCGTATCCAGTGCAGGGTCCGAGGTATTCGCACTGGCTACCT-3'
miR-99a	5'-GTCGTATCCAGTGCAGGGTCCGAGGTATTCGCACTGGCACAAG-3'
U6	5'-AAAATATGGAACGCTTCACGAATTTG-3'
Primers for quantitative Real-time PCR	
miR-99a-up	5'-GCGAACCCGTAGATCCGAT-3'
miR-99a-down	5'-CAGTGCAGGGTCCGAGGT-3'
U6-up	5'-CTCGCTTCGGCAGCACATATACT-3'
U6-down	5'-ACGCTTCACGAATTTGCGTGTC-3'
miR-29a-up	5'-GCGCTAGCACCATCTGAAAT-3'
miR-29a-down	5'-CAGTGCAGGGTCCGAGGT-3'
miR-29b-up	5'-GCGCTAGCACCATTTGAAAT-3'
miR-29b-down	5'-CAGTGCAGGGTCCGAGGT-3'
miR-106a-up	5'-GCGAAAAGTGCTTACAGTGC-3'
miR-106a-down	5'-CAGTGCAGGGTCCGAGGT-3'
miR-124-up	5'- GCGCTAAGGCACGCGGT-3'
miR-124-down	5'-CAGTGCAGGGTCCGAGGT-3'
miR-155-up	5'-GCCGTTAATGCTAATCGTGAT-3'
miR-155-down	5'-CAGTGCAGGGTCCGAGGT-3'

Supplementary Table 2: The miRNAs dysregulated upon treated with cisplatin

The miRNAs upregulated	The miRNAs downergulated
MiR-29a, miR-29b, miR-99a, miR-124, miR-125a, miR-200c, miR-27a, miR-106a, let-7a, let-7c, miR-17, miR-30a, miR-19a and miR-130b	MiR-106a, miR-155, miR-375, miR-299, miR-455 and miR-424
